# Individual attentiveness in vector control should be strengthened during and after the COVID-19 pandemic

**DOI:** 10.3389/fpubh.2022.1055509

**Published:** 2022-11-02

**Authors:** Sulistyawati Sulistyawati, Tri Wahyuni Sukesi, Herman Yuliansyah, Arfiani Nur Khusna, Surahma Asti Mulasari

**Affiliations:** ^1^Faculty of Public Health, Universitas Ahmad Dahlan, Yogyakarta, Indonesia; ^2^Department of Informatics, Faculty of Industrial Technology, Universitas Ahmad Dahlan, Yogyakarta, Indonesia

**Keywords:** COVID-19, dengue, community resilience, vector control, awareness

COVID-19, discovered for the first time in Wuhan, China, at the end of 2019, has spread widely worldwide and has been declared a global pandemic. Due to the infectious nature of the disease, the WHO takes preventive measures to limit its spread by limiting human movement and interaction, which should be implemented thoroughly in all countries (1). This policy has a positive impact on reducing the spread of COVID-19. Still, it harms the control of other diseases that have traditionally been carried out through direct human-to-human interaction, such as dengue prevention. The COVID-19 control policy has at least two implications for dengue vector control in Indonesia, (1) the social distancing policy makes larva monitoring activities that were originally carried out by meeting resident's door to door unable to work, and (2) budget reorientation focused on COVID-19 then make the dengue program not optimal. Accordingly, this opinion emphasizes the importance of strengthened societal capacity in dengue vector control as an implication of COVID-19 disease.

For decades, the dengue prevention program in Indonesia has been carried out through community empowerment, with the larva monitoring cadre going door to door (2). This attempt appears to have usually been running prior to the COVID-19 pandemic due to the raised concerns that dengue outbreaks could occur at any time. So, the dengue prevention program is an effort for early case detection. Several studies have found a link between COVID-19 and the number of dengue cases in some regions (3, 4), including Indonesia. They stated that dengue cases are expected to be lower in 2020 compared to the previous year (4). The Indonesia Ministry of Health predicted that dengue case tends to be lower during the COVID-19 pandemic, as presented in Figure 1 (5, 6). However, this situation must be addressed because it is unclear whether this decrease was caused by mobility restrictions or the public's health-seeking behavior during the pandemic. People are concerned about being diagnosed with COVID-19, so they avoid getting medical checking.

**Figure 1:**
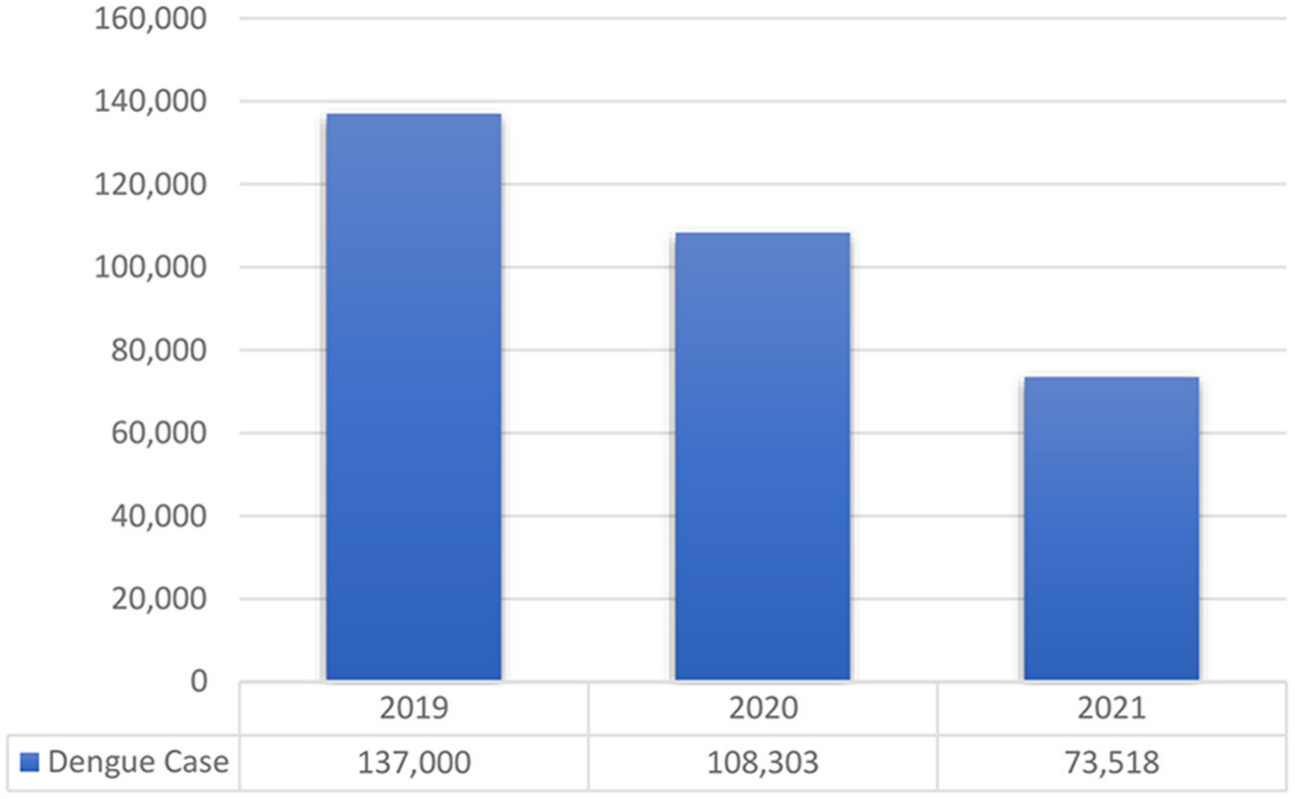
Dengue case in Indonesia 2019–2021.

Since its discovery in Indonesia in 1968, the government has prioritized dengue case management through dengue vector control. The program was carried out by prioritizing observation and direct action at the in-household level, which included reporting data from field observations that were manually tiered from the bottom to the upper level. Furthermore, to maximize efficiency, the Indonesian government prioritizes community participation in vector control through larva monitoring cadre activity (2, 7, 8). This activity generates larvae indices data, which policymakers use to prioritize dengue control interventions. However, the vector control implemented thus far has proven to be counter-productive when COVID-19 occurs, and restrictions on social interaction must be imposed. As a result, monitoring larvae from house to house cannot be done efficiently. The absence of information on the presence of vectors in the community means that the dengue situation in the community cannot be known, which impacts the health system's preparedness in the event of an outbreak. This is understandable, given that previous research has shown that public awareness of dengue symptoms in some Indonesian communities was insufficient (7).

Individuals play an important role in vector control. The individual is the smallest constituent of social structures in society, and their actions will be a collective to a specific purpose of disease prevention, primarily through active participation in programs. This individual's concern and awareness, on the other hand, are influenced by his perception and knowledge of the specific issue (9, 10). However, their knowledge and attitude will determine their involvement in vector control, as stated in previous studies (11, 12). As a result, improving people's knowledge is essential to strengthen individual capacity. So far, many educational programs have been carried out by the Indonesian government through health promotion on the necessity of individual involvement in dengue vector control. Still, due to the lack of evaluation of the results of these promotions, their effectiveness is unknown. This is because, despite the fact that the vector control program for dengue prevention has been in place for some time, a study in Malang City, East Java, found that only around 30% of the community had good knowledge, while only 3.2% among society had good prevention behavior (13).

This is a consideration that improving individual capacity is required by first assessing the state of knowledge and public awareness, as the community may already be saturated with dengue and the information that goes with it. However, this is a scary situation because, based on experience when COVID-19 attacked, it was demonstrated that the community and policy stakeholders were not standing to deal with the double burden of dengue and COVID-19.

## Author contributions

SS was working on the concept, writing the first draft, and finalizing it. TS, HY, AK, and SM were reviewing and commenting on the draft. All authors contributed to the article and approved the submitted version.

## Funding

This article is part of a larger study funded by the Ministry of Education and Culture of the Republic of Indonesia under grant number 030/PB.PDUPT/BRIn.LPPM/VI/2022.

## Conflict of interest

The authors declare that the research was conducted in the absence of any commercial or financial relationships that could be construed as a potential conflict of interest.

## Publisher's note

All claims expressed in this article are solely those of the authors and do not necessarily represent those of their affiliated organizations, or those of the publisher, the editors and the reviewers. Any product that may be evaluated in this article, or claim that may be made by its manufacturer, is not guaranteed or endorsed by the publisher.
